# Development of basic building blocks for cryo-EM: the *emcore* and *emvis* software libraries

**DOI:** 10.1107/S2059798320003769

**Published:** 2020-03-31

**Authors:** José Miguel de la Rosa-Trevín, Pedro Alberto Hernández Viga, Joaquín Otón, Erik Lindahl

**Affiliations:** a Department of Biochemistry and Biophysics, Science for Life Laboratory, Stockholm University, Stockholm, Sweden; b UNEAC Manzanillo, Cuba; c Medical Research Council Laboratory of Molecular Biology, Cambridge, United Kingdom; d Swedish e-Science Research Center, KTH Royal Institute of Technology, Stockholm, Sweden

**Keywords:** cryo-EM, image processing, data visualization, software development

## Abstract

Image-processing software has always been an integral part of structure determination by cryogenic electron microscopy (cryo-EM). Recent advances in hardware and software are recognized as one of the key factors in the so-called cryo-EM resolution revolution. Increasing computational power has opened many possibilities to consider more demanding algorithms, which in turn allow more complex biological problems to be tackled. Moreover, data processing has become more accessible to many experimental groups, with computations that used to last for many days at supercomputing facilities now being performed in hours on personal workstations. All of these advances, together with the rapid expansion of the community, continue to pose challenges and new demands on the software-development side. In this article, the development of *emcore* and *emvis*, two basic software libraries for image manipulation and data visualization in cryo-EM, is presented. The main goal is to provide basic functionality organized in modular components that other developers can reuse to implement new algorithms or build graphical applications. An additional aim is to showcase the importance of following established practices in software engineering, with the hope that this could be a first step towards a more standardized way of developing and distributing software in the field.

## Abstract 2

This article presents an overview of the development of two basic software libraries for image manipulation and data visualization in cryo-EM: *emcore* and *emvis*.

## 1. Introduction   

Advances in cryo-EM have been closely related to image-processing and software development since the early days of the field (Kühlbrandt, 2014 ▸; Belnap, 2015 ▸; Bai *et al.*, 2015 ▸). Back in 1992, seven software packages that were in use at the time were reviewed by Hegerl (1992 ▸), and just a few years later, in 1996, a special issue of the *Journal of Structural Biology* was dedicated to software tools for molecular microscopy (Carragher & Smith, 1996 ▸). Over time, the number of programs has increased considerably. At the time of writing, the Wikipedia page https://en.wikibooks.org/wiki/Software_Tools_For_Molecular_Microscopy contained 17 general packages, 27 specific packages, 38 application tools, 19 visualization tools and six utilities, with many programs not being registered on the list.

Only considering packages focused on single-particle analysis, there are a myriad of tools available to the community. These range from command-line programs to complete software suites (or packages) in which the whole processing workflow can be executed. Several of these packages have been under development and improved over many years or decades, accumulating algorithms and methodologies for cryo-EM image processing. Nonetheless, from a software-design point of view, there is room for much more improvement. Most of the development of existing software has been focused on the scientific part, while the overall architectural design has not been a priority. Currently, there is a large amount of functionality that is redundant among existing packages and the major way of reusing other code is by duplicating it from its original source. This approach is a waste of energy for new development and is not sustainable in the long term for the field. In his review in 1992, Hegerl was already raising the concern about consolidating existing packages into a modular system supported by the entire community (Hegerl, 1992 ▸). The same idea of a common library was the main driving force for the design and implementation of the *Image Processing Library and Toolbox* (*IPLT*; Philippsen *et al.*, 2007 ▸), which grew into a full package but does not seem to be under active development. Despite the clear advantages of a common effort in terms of software libraries, there are many complex factors that should be taken into account to analyze the reasons for such an initiative to be widely adopted (or not) by the community (Smith & Carragher, 2008 ▸). One example of a basic library that has proven to be useful is *mrcfile* (Burnley *et al.*, 2017 ▸), which provides functions to operate with MRC image files.

In this work, we present the design and implementation of two basic software libraries for cryo-EM: *emcore* and *emvis*. During their development, great care has been put into the overall design of the application programming interface (API) in order to provide other developers with basic building blocks. *emcore* contains classes and methods to operate with image data and metadata, while *emvis* implements graphical components for data visualization and analysis. In the following, we will discuss the key points of both libraries, highlighting design decisions and possible applications. Finally, we will discuss future directions of the current work and general conclusions.

## 2. Design and implementation   

### 2.1. Architecture overview   

One of our core principles when conceiving these libraries has been modularity. This is the main reason why all of the code has not been concentrated into a single library with many dependencies. Usually, graphical components bring more dependencies into a project that are not needed by command-line programs or if other graphical implementations are considered. For both *emcore* and *emvis* we have chosen to follow object-oriented programming paradigms, which have been implemented in C++ (Stroustrup, 2000 ▸) and Python (https://www.python.org/). This combination is quite common in scientific domains, since C++ allows more low-level control and more efficient code, while Python complements it with its high flexibility and elegant syntax. Both languages are widely used and have a variety of supporting libraries for general-purpose and scientific computing.

**Figure 1:**
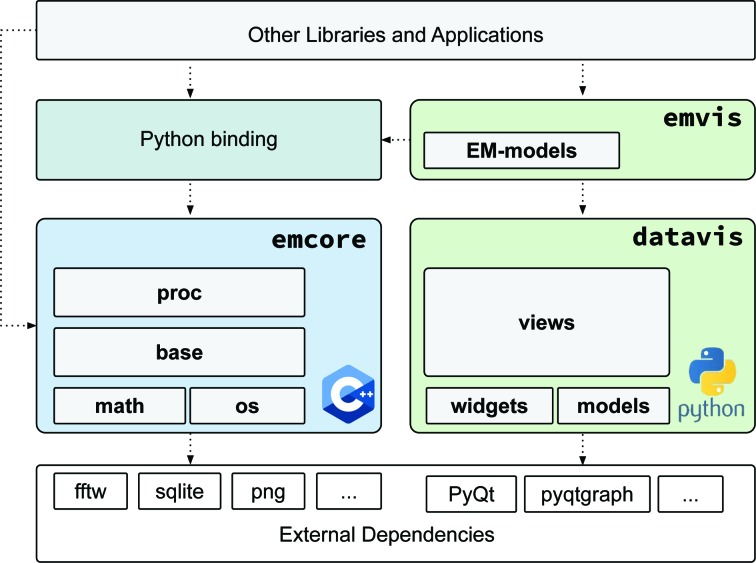
General architecture overview showing different components and their interaction. Dotted arrows represent dependencies from one module to another. *emcore* is a C++ library with basic functions for image and table manipulation. A binding layer provides access to *emcore* from libraries or applications written in Python. This binding is used by *emvis* to implement visualization components, based on general models, views and widgets defined in *datavis*.

The overall software-architecture layout is depicted in Fig. 1 ▸. The *emcore* library contains submodules for basic mathematical operations and operating system-specific functions. The *base* submodule supports the essence of cryo-EM data handling: Images and Tables. Based on these submodules, the processing submodule defines ImageProcessor as the base class to implement basic operations such as filters, alignments, Fourier transforms *etc*. *emcore* is implemented in C++11, exploiting some of the new features of this version for more efficient memory manipulation and more standard library functions. Dependencies from external libraries are centralized and some of them are even optional (for example, libraries for reading specific types of images or tabular formats). Additionally, we use *pybind*11 (Jakob *et al.*, 2017 ▸) to generate a binding layer to the existing classes and methods that exposes most of the available functionality to Python. The general architecture, as well as many elements of the design and implementation of the main components, has been inspired and influenced by existing code in established packages such as *Xmipp* (Sorzano *et al.*, 2004 ▸; Scheres *et al.*, 2008 ▸; de la Rosa-Trevín *et al.*, 2013 ▸), *EMAN*2 (Tang *et al.*, 2007 ▸), *Bsoft* (Heymann & Belnap, 2007 ▸) and *RELION* (Scheres, 2012 ▸; Kimanius *et al.*, 2016 ▸; Zivanov *et al.*, 2018 ▸), among others. Nonetheless, a strong emphasis has been put on the clear definition of each class API, separating the implementation details from the public interface that is visible to other developers.

On the other hand, *emvis* has been written entirely in Python, where the more general components are grouped into a separate library, *datavis*. This library contains definitions of the data models that will be used by the graphical View components. Some utility widgets are implemented using PyQt5 (https://www.riverbankcomputing.com/software/pyqt/intro) and PyQtGraph (Luke, 2011 ▸). Even if the initial focus is cryo-EM data processing, the *datavis* design is more general and does not depend on *emcore*, opening the possibility for it to be reused in other applications dealing with images and metadata. On top of *datavis*, the *emvis* library implements some of the defined models more specifically for cryo-EM data using the Python binding provided by *emcore*.

### 2.2. Using *emcore* and *emvis*   

**Figure 2:**
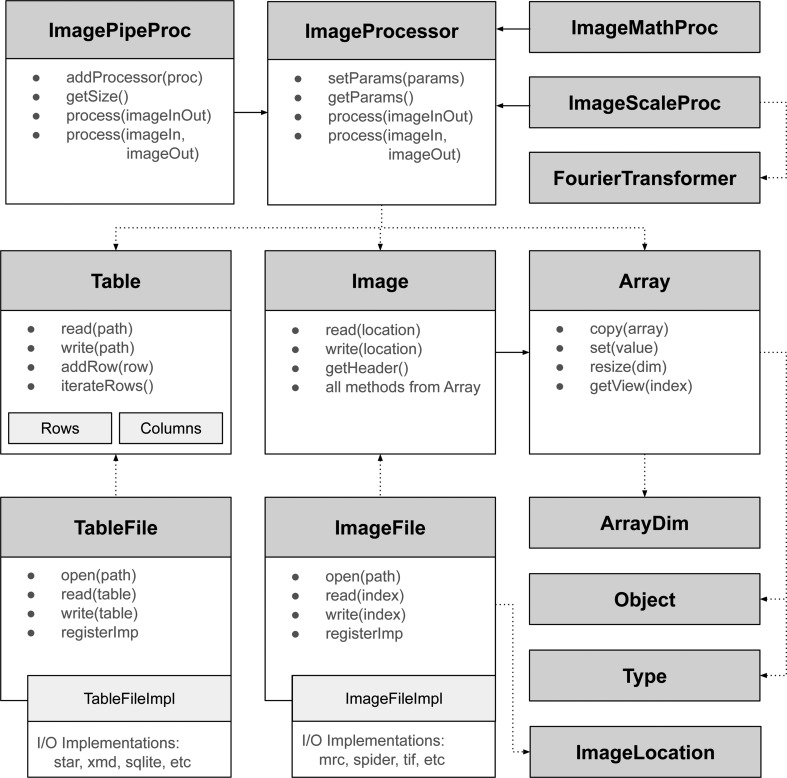
Simplified class diagram of the *emcore* library, where dotted arrows represent dependencies and solid arrows represent inheritance. Image and Table are the main classes used for data manipulation and rely on other basic classes such as Array, Object and Type. Input/output operations are delegated to separate classes, which can be extended to support other formats. The abstract definition of ImageProcessor allows the implementation of many image-processing operations such as arithmetic or windowing/scaling. The FourierTransformer class is used in the many-processors implementation. The special ImagePipeProc allows the combination of many processors to perform a given operation.

The *emcore* library is built around three main classes: Image, Table and ImageProcessor. Image and Table are the basic units for data handling that separate how data are represented in memory from how data are read/written from different formats. This approach simplifies the task of implementing support for new formats in the future. Image­Processor is the base class that defines the general interface for all processing operations. All entities rely on more basic utility classes such as Type, Object and Array, as shown in Fig. 2 ▸.

The ImageFile class allows images to be read and written from/to individual files or stacks. Internally, this class selects the implementation to deal with a specific image format (for example MRC, SPIDER, TIF *etc*). The format implementation class will then handle the supported data types (for example float, int8, double *etc.*) and will perform internal type conversions if required. Binary image data are then stored by the Image class, which is implemented as a four-dimensional array structure. The Image class is independent of file format, although it contains convenience read and write methods (internally using an ImageFile) for simple input/output operations.

**Figure 3:**
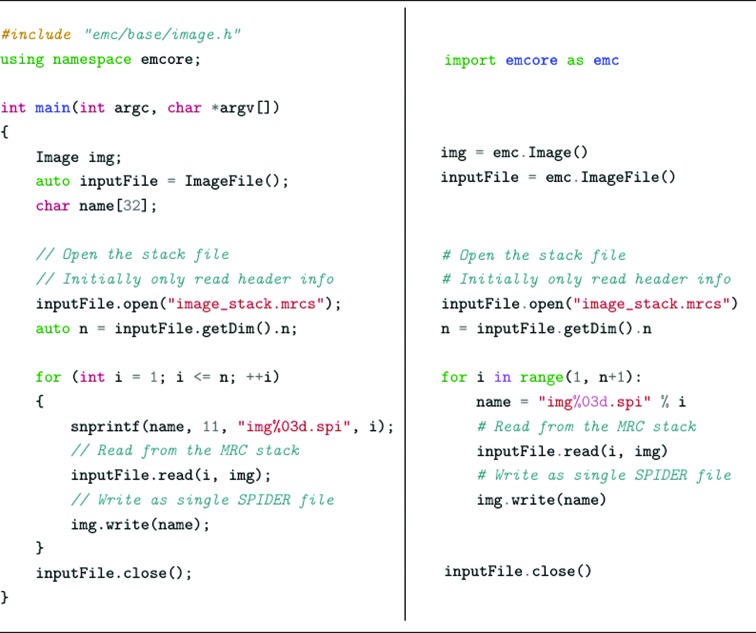
Code example showing how to use the Image and ImageFile classes to read from one image format and write to another. The left side is C++ code and the right side is the Python equivalent.

The code listing in Fig. 3 ▸ shows how an image stack in MRC format is opened (the MRC implementation is instantiated internally by the ImageFile class) and each image is written into a separate file in SPIDER format. It can be seen that the code in both C++ (left) and Python (right) is very similar, apart from syntactic differences between the languages.

Together with the Image class we also implemented a Table class, in which the internal data structure is also separated from how it is read/written to disk. The Table class allows tabular data to be manipulated by operating on columns or data rows. Similarly to the Image case, the TableFile class will centralize the implementation to read/write-specific file formats (for example, STAR, XML, SQLITE *etc*). Support for other formats can be added to the library without affecting the way these classes are used.

On top of the classes mentioned above, the ImageProcessor class defines the primary interface for implementing new image-processing operations (see Fig. 2 ▸). Each processor can define variable parameters which are essentially a dictionary of Object values. Apart from these parameters, there is a process() function that takes an image as input and modifies it in place or stores the result as an output. For example, ImageMathProc implements some basic arithmetic operations, while Image­ScaleProc performs resizing/scaling using the Fourier­Transformer class, which is a thin wrapper around the FFTW library (Frigo & Johnson, 2005 ▸).

**Figure 4:**
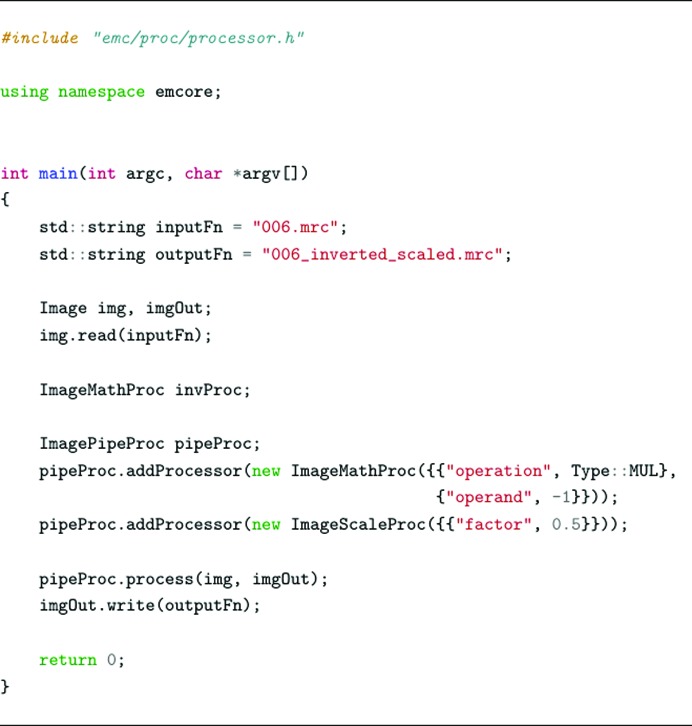
Simple code example using the ImagePipeProc class. First an ImageMathProc is used to invert the pixel values (multiplying by −1) and the image is then scaled half of its size using ImageScaleProc.

ImagePipeProc is a special type of ImageProcessor that allows the concatenation of several other processor objects. It has a special function addProcessor() that allows a new processor to be added to the internal ‘processing pipeline’. Then, a call to its process() function will execute all of the internal processors to produce the desired result. Intermediate image objects are handled by the ImagePipeProc class and this generic implementation opens the possibility of future performance improvements such as parallel execution of the pipeline. The code in Fig. 4 ▸ shows a simple example of using ImagePipeProc to create a processor that multiplies the pixel values by −1 and scales the image by half of its original size.

Visualization is an important component of cryo-EM data analysis; therefore, from the beginning we started developing the *emvis* visualization library using *emcore* as a backend. As shown previously in Fig. 1 ▸, there is a separate *datavis* library that contains more general components (mainly for image and table display), with *emvis* being more specific for cryo-EM data. *datavis* is composed of three main submodules: *models*, *widgets* and *views*.

The *models* submodule contains the definitions of several data models that will be used by graphical view components. Some models are related to binary data, while others are related to tabular data. TableModel is used by the graphical components to query the data structure such as the existing columns or the number of rows. On the other hand, TableConfig allows different visualization configurations to be specified based on the same data model. For example, it is possible to display only some columns, render a given data column or specify which labels will be displayed together with images. This flexibility allows developers to avoid writing repetitive code, while still allowing them to present the data contextually.

In the *widgets* submodule we have implemented some simple components with very specific functionality. Based on both *models* and *widgets*, the *views* submodule defines higher level graphical components that help to visualize more complex data. Furthermore, the *emvis* library implements some extra views that are more specific for cryo-EM data formats. The provided graphical classes will help developers to quickly create simple applications while minimizing the required knowledge about the underlying PyQt5 toolkit. Some concrete applications of the *emvis* components are described in the next section.

### 2.3. Applications   

In this section, we mention some of the already developed applications of the library. These applications exemplify how to use some of the underlying classes and graphical components. A more detailed list of currently implemented tools can be found at https://3dem.github.io/emdocs/emvis/.

#### 2.3.1. Command-line tools   

While developing these libraries, two command-line tools were developed to help during implementation and testing. *em-image* is one of these programs and provides some functions for image manipulation. It contains basic operations similar to those provided by other existing packages, but we find it convenient to include such a tool for command-line operations without the need to write code. One interesting feature of the program is that many operations can be concatenated and applied to the input images without requiring the writing of intermediate files, as is performed by many other implementations. Another provided program is *em-table*, which deals with tabular data. It supports many operations to analyze and modify data items. Although it is also possible to find similar tools in other packages, this program provides some advanced operations that are usually achieved in more cumbersome ways using text processors such as AWK. A complete description of these programs together with many examples can be found at https://3dem.github.io/emdocs/emcore/.

#### 2.3.2. File browser   

**Figure 5:**
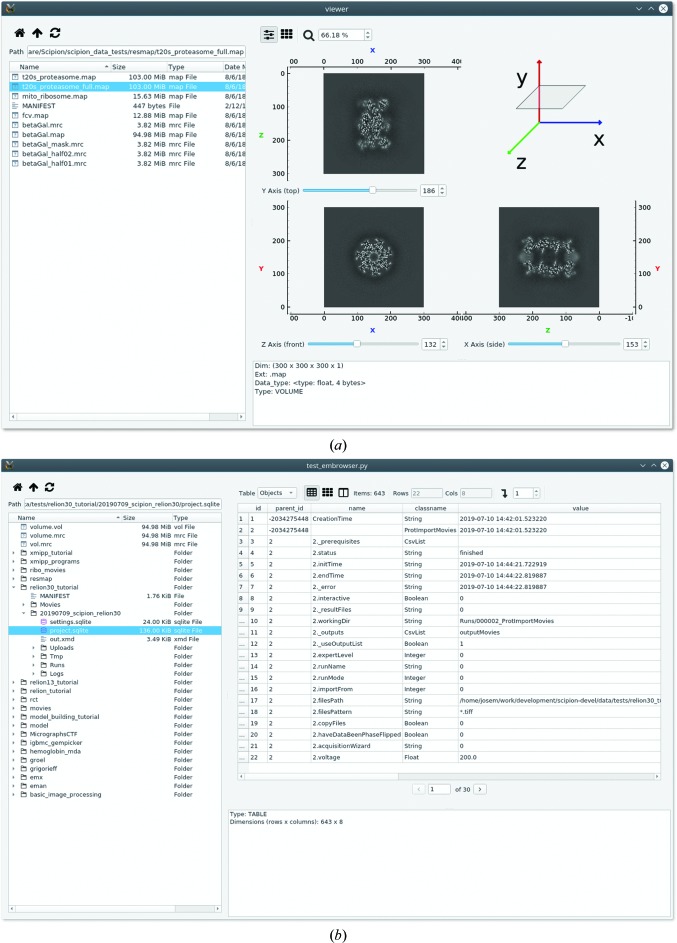
Screenshots of different data visualizations with BrowserView. (*a*) Navigation in ‘Directory’ mode, where a volume is selected and previewed with MultiSlicesView. (*b*) Navigation in ‘Tree’ mode, which displays a Sqlite3 file generated by *Scipion*.

We have also implemented a file browser that uses the tools provided in *emvis*. It has turned out to be quite a useful application that helps to navigate the output results from existing programs such as *RELION* (Scheres, 2012 ▸; Kimanius *et al.*, 2016 ▸; Zivanov *et al.*, 2018 ▸), *Scipion* (de la Rosa-Trevín *et al.*, 2016 ▸) and others. The browser itself is a component that could be embedded in other applications. When exploring files, the browser uses the factory classes in *emvis* to create appropriate views of the selected file, which are displayed in the preview panel. For example, Fig. 5 ▸(*a*) shows the MultiSlicesView for the selected volume and Fig. 5 ▸(*b*) shows the ColumnsView for a Sqlite3 (https://www.sqlite.org) file generated from *Scipion*. The left panel shows the list of files and supports two navigation modes: one in which a file in the current folder is shown (Fig. 5 ▸ *a*) and another that expands as a tree view (Fig. 5 ▸ *b*). The browser can be invoked from the command line by using the *em-viewer* program with a folder path as its first argument.

#### 2.3.3. Particle-picking visualization   

**Figure 6:**
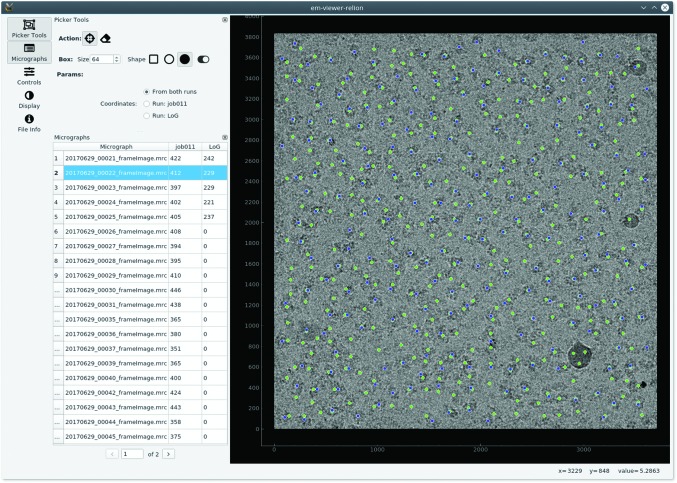
Screenshoot of the *em-picker* application comparing two different results from *RELION* particle-picking runs.

Another versatile component is PickerView, implemented in *emvis*, which is instantiated by the *em-viewer* program to display the results of particle picking. The underlying PickingModel allows the easy support of different output formats from many programs such as *Xmipp* (Sorzano *et al.*, 2004 ▸; Scheres *et al.*, 2008 ▸; de la Rosa-Trevín *et al.*, 2013 ▸), *RELION* (Scheres, 2012 ▸; Kimanius *et al.*, 2016 ▸; Zivanov *et al.*, 2018 ▸), *Scipion* (de la Rosa-Trevín *et al.*, 2016 ▸), *EMAN* (Tang *et al.*, 2007 ▸), *crYOLO* (Wagner *et al.*, 2019 ▸) and *Topaz* (Bepler *et al.*, 2019 ▸). It also facilitates the addition of new programs by providing a minimal amount of code to parse from a specific format. Fig. 6 ▸ shows a screenshot of a tool comparing two picking results. In this example, it is comparing outputs from two *RELION* picking runs, but it can easily be adapted to compare different picking programs. Additionally, a mask-creator tool has been integrated with *MicrographCleaner* (Sanchez-Garcia *et al.*, 2019 ▸), highlighting possible bad regions from the micrographs and particles lying in these regions.

## 3. Code availability, installation and documentation   

We strongly believe that open-source software is one of the supporting pillars of modern science, and as such we distribute the tools described in this work under GPLv3 (https://www.gnu.org/licenses/gpl-3.0.en.html). Apart from the technical aspects, another strong motivation for this work is to create a community project that will unite efforts among scientific developers. The only possible way to pursue this goal is to use a license that protects the freedom of the code and encourages collaboration and sharing.

Source code is freely available at the following repositories: *emcore* at https://github.com/3dem/emcore, *datavis* at https://github.com/3dem/datavis and *emvis* at https://github.com/3dem/emvis.

Regarding installation, a large amount of effort has been dedicated to simplifying the installation process and distributing the code as standard Python packages. The three libraries can be installed into an existing Python environment using pip by the command pip install emcore datavis emvis or, using Conda, conda install emcore datavis emvis -c conda-forge -c emforge.

Another priority of this work is to produce good documentation around the provided code. Again, engaging the community to contribute is very important, and for this documentation is essential to growing and sustaining the community. Good documentation helps prospective users to succeed using the software and enables them to give further feedback.

Online documentation can be found at https://3dem.github.io/emdocs/emcore/ for *emcore* and at https://3dem.github.io/emdocs/emvis/ for *datavis* and *emvis*.

## 4. Conclusions and future plans   

Here, we have presented our work in developing basic libraries for cryo-EM image processing and data analysis. The main focus of these libraries is to create solid building blocks with a clear API and following good software-engineering practices. Currently, the libraries do not aim to implement many processing algorithms or visualization tools, but rather to establish a well structured framework that others might build on. Nonetheless, the current implementation already provides interesting features that could be useful for users or developers. Moreover, the overall design has been conceived with integration in mind, either with other cryo-EM packages in the field or with more general scientific packages in the Python ecosystem.

Even if we have made the current design and implementation thinking of the future and of possible integration, we have also identified existing cases where this work may already be useful. For example, the *Scipion* framework will benefit from using the provided libraries to rewrite some of the core functionality and some of the visualization tools. This approach will remove the current dependencies on the more complex *Xmipp* software package, and will fit better with the current *Scipion* philosophy of a general Python framework with modular plugins. Nonetheless, some legacy classes have been included to facilitate possible migrations from existing C++ codebases such as *Xmipp* or *RELION*. While we understand the amount of work that is involved in a nontrivial refactoring process, it may be that a progressive, partial incorporation of the libraries could be considered.

We still expect to polish some details of the current implementation with feedback from the community, but do not anticipate major changes in the overall design and architecture. A possible extension of the current work would be to incorporate basic functions for parallelization with minimal effort. For example, some options that could be implemented include threading, multiprocessing and graphics cards. By having some ready-to-use built-in functions, scientists could prototype ideas more quickly and decide whether it is worth investing effort in further optimization.

It is almost impossible to predict the success of these libraries in the future and whether they will be widely adopted by the community. We have tried our best to provide easy-to-use, extensible and robust code to remove possible hurdles, but there are many factors beyond technical aspects. We hope that this work will lay the first stone for building a more collaborative environment for software development in cryo-EM, where best practices are shared and best ideas are consolidated.
